# Twenty years of transposable element analysis in the *Arabidopsis thaliana* genome

**DOI:** 10.1186/s13100-020-00223-x

**Published:** 2020-07-27

**Authors:** Hadi Quesneville

**Affiliations:** Université Paris-Saclay, INRAE, URGI, 78026 Versailles, France

## Abstract

Transposable elements (TEs) are mobile repetitive DNA sequences shown to be major drivers of genome evolution. As the first plant to have its genome sequenced and analyzed at the genomic scale, *Arabidopsis thaliana* has largely contributed to our TE knowledge.

The present report describes 20 years of accumulated TE knowledge gained through the study of the *Arabidopsis* genome and covers the known TE families, their relative abundance, and their genomic distribution. It presents our knowledge of the different TE family activities, mobility, population and long-term evolutionary dynamics. Finally, the role of TE as substrates for new genes and their impact on gene expression is illustrated through a few selected demonstrative cases. Promising future directions for TE studies in this species conclude the review.

## Introduction

Transposable elements (TEs) are mobile, repetitive DNA sequences that constitute a structurally dynamic component of genomes. They have been extensively studied in plant genomes, and have been shown to be an important source of variation on which natural selection can operate to evolve species, or agronomical selection to obtain interesting varieties [1].

As the first plant genome sequenced and analyzed at a genome scale, *Arabidopsis thaliana* has largely contributed to this knowledge. As a model species, the wealth of diverse data accumulated over the years has provided incomparable tools for understanding TE biology in *Arabidopsis*. This accumulated knowledge can serve today as a baseline to understand TEs in other plants that may be relevant for crop improvement. However, note that small genomes such as *Arabidopsis thaliana* may substantially differ from very large plant genomes in terms of TE dynamics and impact on host genome.

In this review I report the main findings on TE biology that make the plant model species *Arabidopsis thaliana* a major contributor to our current knowledge of plant TEs. This is the result of successive rounds of analysis of the reference genome over 20 years, incrementally improving our understanding as new tools or new data sets became available. Today, thanks to continuous advances in sequencing technology, the resequencing of hundreds of natural accessions completes our view of TE landscape in the species. This review presents an overview of the TE families that have been found, their dynamics at the species level, their impact on host genes, and how they contribute to genome evolution.

## Transposable element families in *Arabidopsis thaliana*

### Known types of transposable element families

TEs are classified according to their transposition mechanism [2]. Class I TEs transpose through an RNA intermediate with a “copy and paste” mechanism, and are also called retrotransposons. This category is further subdivided into orders. The LTR retrotransposon order contains sequences with (i) Long Terminal Repeats (LTR), (ii) a polyprotein *pol* that encodes a protease, a reverse transcriptase, a ribonuclease H, and an integrase, providing the enzymatic machinery for reverse transcription and integration into the host genome, and (iii) a *gag* gene that encodes a viral particle coat. Sometimes an envelope-like gene (*env*-like) can be found. The presence of the *env*-like gene raise interesting discussions when discovered in plants with the speculation that these elements may be retroviruses [3–5]. Indeed, a functional role of an envelope protein for viral propagation in a plant is unknown as cell walls preclude membrane fusion. However, the presence of *env* genes in plant viruses is not unusual. Depending on the TE, two different mechanisms can be used to express the *gag* and the *pol* genes: a fusion into a single open reading frame (ORF) that is then cleaved, or the introduction of a frameshift between the two ORFs. This frameshifting allows the production of both proteins, while ensuring that much more *gag* protein is produced to form virus-like particles.

The LTRs of retrotransposons are divided into three functional areas: U3, R, and U5 [6]. U3 may contain regulatory motifs and promoter region at the 3′ end, and R contains both the start and termination sites for transcription. Most elements have the terminal dinucleotides 5′-TG…CA-3′ which tend to be part of larger terminal inverted repeats. LTR retrotransposons have a potential tRNA primer-binding site, downstream of the 5′ LTR, recognized as a short polypurine sequence termed primer-binding site (PBS). This 10–20 nucleotide sequence can partly base-pair with a tRNA molecule. The sequence near the LTR termini that begins with 5′-TGG-3′ is complementary to the CCA trinucleotide present at the 3′ end of all tRNAs and is a feature of all PBSs that utilize the 3′ end of a tRNA as a primer [7]. A short purine-rich sequence near the 3′ LTR, called polypurine track (PPT), primes the second strand DNA synthesis for the retroviral transposition cycle. The LTR retrotransposon order is further subdivided into superfamilies to distinguish Copia and Gypsy according to the position of the integrase in the polyprotein *pol*. Some defective copies may transpose if they have conserved the two LTR as well as the PPT and the PBS signals.

The non-LTR retrotransposons have no LTR at their extremities. They are subdivided into the LINE and SINE orders. LINEs encode (i) an endonuclease, which makes a single-stranded nick in the genomic DNA, and a reverse transcriptase, which uses the nicked DNA to prime reverse transcription, and (ii) a non-sequence-specific RNA binding protein that contains zinc finger, leucine zipper, and coiled-coil motifs and functions as chaperone. LINEs are terminated by a polyA or A/T-rich 3′ tail. Whereas most TEs are transcribed by RNA polymerase II for their transposition, SINEs are transcribed by RNA polymerase III as they generally evolved from t-RNA genes (sometimes 7SL in mammals). Similar to tRNA genes, SINE sequences contain two well-conserved motifs, called box A and box B, that serve as an internal promotor for transcription of the element by RNA polymerase III. SINEs do not encode any protein, but are mobilized by LINE machinery in *trans*. The 5′ region of SINEs is similar to tRNA genes [8] while the 3′ region of many SINEs shows similarity to the 3′ end of LINEs. Other types of retrotransposons have not been described so far in *Arabidopsis*.

**Fig. 1:**
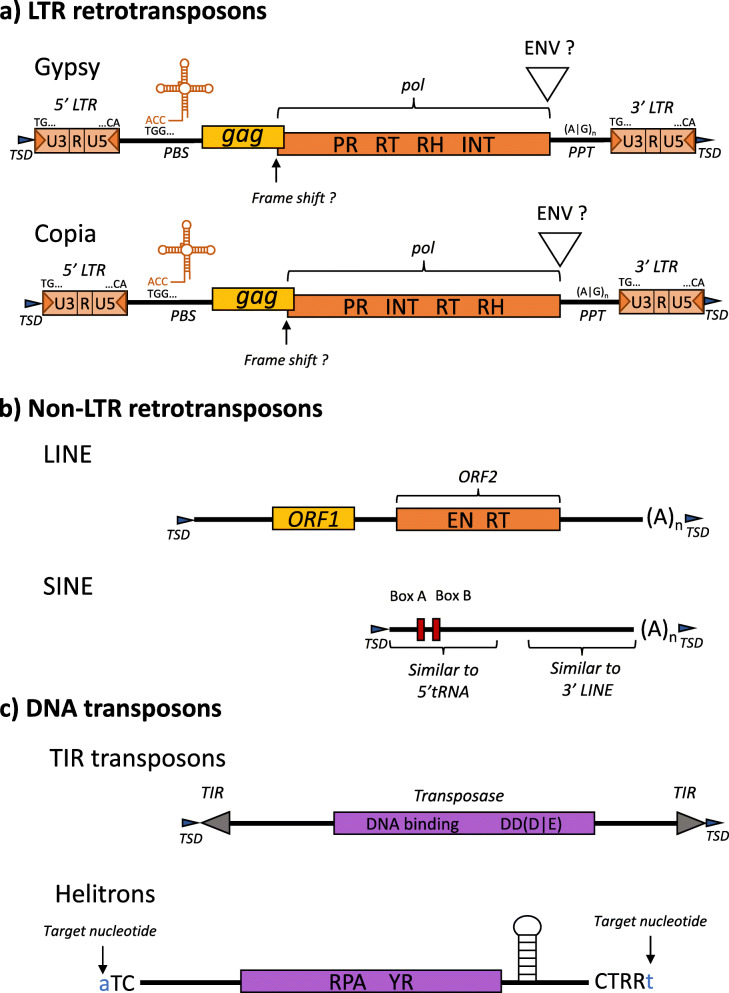
Transposable element structures. **a)***LTR retrotransposons: gag* encodes a polyprotein with a capsid and a nucleocapsid domain. The Pol gene produces three proteins: a protease (PR), a reverse-transcriptase endowed with an RT (reverse-transcriptase) and an RNAse H domain, and an integrase (INT). The *gag* and *pol* genes are expressed either as a fusion into a single open reading frame (ORF) that is then cleaved, or by the introduction of a frameshift between the two ORFs. Sometimes an envelope gene (ENV) is found. LTRs are divided into 3 regions: U3 may contain regulatory motifs and a promoter region, R contains both the start and termination sites for transcription, and U5 is the remaining part. The PBS operates as tRNA primer-binding to prime the first strand DNA synthesis, the minus-strand. The PPT is a short purine-rich sequence that primes the second strand DNA synthesis, the plus-strand. *TSD:* Target Site Duplication is a short direct repeat that is generated on both flanks of a TE upon insertion. Gypsy and Copia superfamilies differ according to the position of the integrase in the polyprotein *pol.***b)***LINE*: Encodes two open reading frame: ORF1 and ORF2. ORF1 encodes a non-sequence-specific RNA binding protein that functions as chaperone. ORF2 encodes an endonuclease (EN), which makes a single-stranded nick in the genomic DNA and a reverse transcriptase (RT), which uses the nicked DNA to prime reverse transcription. They are terminated by a polyA or A/T-rich tail. *SINE*: SINE sequences contain the box A and B conserved motifs that serve as an internal promotor. **c)***TIR*: TIR transposons encode a transposase protein necessary for mobility, and are bordered by Terminal Inverted Repeats (TIRs). *Helitrons:* generally contain a Y2-type tyrosine recombinase (YR) along with Replication protein A (RPA) and other proteins to catalyze their mobility, a hairpin structure, 5′ TC and 3′ CTRR termini (R = A or G). Insertion occurs into the target dinucleotide AT (shown in lowercase)

Class II TEs, or DNA transposons, transpose as a DNA molecule. The TIR order TEs move using a “cut and paste” mechanism. They encode a transposase with a DDE or DDD domain, the protein allowing the mobility, and are bordered by an inverted repeat. The Helitrons constitute another order which transpose through a putative rolling circle mechanism that remains obscure. They generally contain a Y2-type tyrosine recombinase along with some other proteins to catalyze their mobility, and a hairpin structure in the second half of the sequence. Their 5′ are terminated by the 5′-TC-3′ nucleotides and the 3′ by a 5′-CTRR-3′ degenerate sequence (R stands for A or G). Helitrons insert into the target dinucleotide AT. TIRs and Helitrons both have defectives elements that can be mobilized in *trans* by complete copies. Note that Helitrons are often mis-annotated as gene or simply missed when defectives as they are difficult to caracterised when truncated. The two remaining known types of DNA transposons, Cryptons and Mavericks, have not been described so far in *A. thaliana*. Figure 1 show the sequence structures of the different superfamilies. Table 1 summarizes TE categories and their respective family numbers found in *Arabidopsis* [9].

**Table 1:** Arabidopsis TE categories

Classes	Orders	Superfamilies	Family numbers
Class I	LTR retrotransposons	Copia	109
		Gypsy	32
	LINE	L1	11
		Unknown	1
	SINE	t-RNA	5
Class II	TIR	En-Spm	12
		MuDR	70
		Harbinger	3
		hAT	22
		Pogo	4
		Mariner	2
		Tc1	1
		Unknown	12
	Helitron	Helitron	34

The superfamily having the highest number of families are Copia (109) followed by MuDR (70), Helitron [10], Gypsy [11] and hAt [12]. All other superfamilies have fewer than 11 families.

### Relative abundance of each major type in the genome

The very first analysis reported that TEs account for at least 10% of the genome, or about one-fifth of the intergenic DNA [13]. Since this pioneer study, other authors provided improved reannotation of TE content using more and more sensitive approaches [14–17]. Current official annotation available in TAIR10 indicates that TEs account for ~ 21% of the genome [14].

**Fig. 2:**
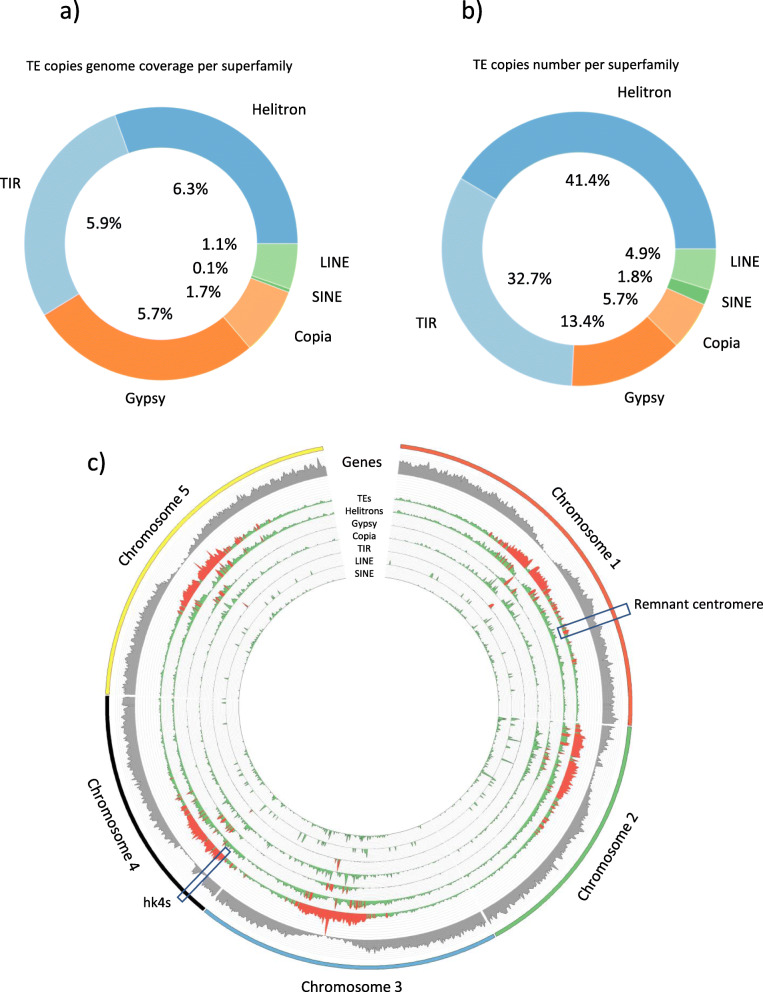
TE genomic distributions. **a)** TE category genome coverage. **b)** Relative TE category copy numbers. **c)** Chromosomal copy number distribution per 100 kb windows overlapping by 10 kb. Red bars indicate regions with more than 30 copies in the window

Currently, the reference genome of *Arabidopsis thaliana* is 125 Mb long and contains ~ 32,000 TE copies, generally truncated and degenerated, that belong to 318 families [9]. Retroelements represent the largest fraction of TE sequences (10 Mb), followed by Helitrons (8 Mb) and DNA transposons (7 Mb). Figure 2a shows genome coverage of the TE categories, and Fig. 2b shows relative abundance per category (from Ahmed et al. 2011 [9], supplementary data).

**Fig. 3:**
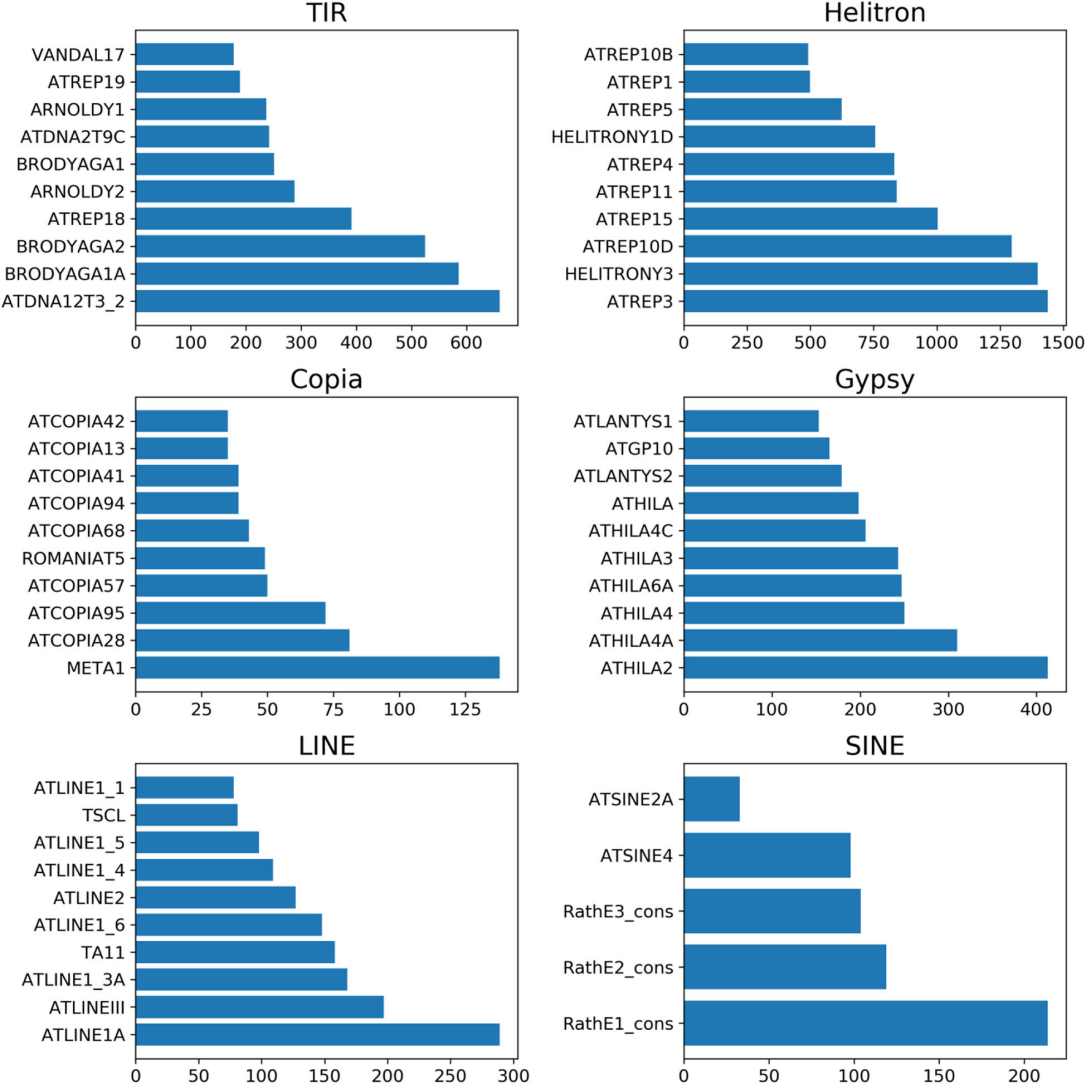
Ten most abundant TE families per TE category

The most abundant TEs are Helitrons with four families with more than 1000 copies: ATREP15 (1003 copies), ATREP10D (1295 copies), HELITRONY3 (1399 copies), ATREP3 (1439 copies). DNA transposons follow, with BRODYAGA2 (525 copies), BRODYAGA1A (586 copies), and ATDNA12T3_2 (660 copies) being the most abundant. LTR retrotransposons have fewer copies, ATHILA2 (413 copies) and ATHILA4A (310 copies) having the highest copy numbers. LINEs are less frequent, the most present are ATLINEIII (197 copies) and ATLINE1A (289). The most abundant SINE is RathE1_cons (214 copies). Figure 3 shows the 10 most abundant families per category (from Ahmed et al. 2011 [9], supplementary data).

## Chromosomal distribution

Figure 2c shows chromosomal distributions for genes, TEs, and by TE categories. Nucleotide content in pericentromeric regions is dominated by LTR retrotransposons, in particular the *Gypsy* superfamilies with 70.7% of their copies in the heterochromatin [15]. Several authors show this clear higher abundance of LTR retrotransposons in the heterochromatin [17, 18].

For all other TE sequences, with the exception of *En-Spm*, a small bias of accumulation in euchromatin relative to heterochromatin is observed [15]. This is supposed to be the result of faster elimination of TE sequence in heterochromatin due to frequent ectopic recombination between repeats present in high density in this compartment, coupled with weak selective constraints in this gene poor region.

In addition to the pericentromeric regions, a knob (an interstitial heterochromatic region) called *hk4S* is well-known in *Arabidopsis thaliana* accession *Col0*. It represents a recent formation through an inversion and shows a TE distribution profile intermediate between those of the euchromatin and the heterochromatin. Few TE superfamilies are represented in the *hk4S*, most TEs in this region are members of the highest copy-number families (e.g., *Helitron*, LINE, *Gypsy, Copia* and *MuDR*).

Remarkably, the deep TE annotation unveils the presence of new regions of high repeat content beyond those defined by the pericentromeres and the knob, especially one peak on chromosome 1 [19, 20]. Ancestral genome reconstruction shows that this chromosome is a fusion of two ancestral chromosomes and this high density repeat region may correspond to the ancestral centromere of one of the fused chromosomes.

## Transposable element activity

### Transposable element ages

The TE sequence identity with respect to the consensus sequence used for annotation, is related to the age of the TEs. High identity scores indicate sequences that have accumulated very few mutations since their divergence from their common ancestor and therefore can be considered to be young. Sequence identity varied between 58 and 100% with a mean at 80.4% [15]. Mean identity did not differ between the heterochromatic and the knob TE sequences (mean sequence identity: 82%), but euchromatic TE sequences had a statistically significant lower value (mean sequence identity: 80%) consequently appearing older. Overall, this suggests that most TE families are quite old in this genome, probably appearing in a common ancestor of the *Brassicaceae* lineage. However, despite the old origins of *Arabidopsis thaliana* TEs, half of the 326 families annotated show recent transposition events, as suggested by the polymorphism found in a study of 211 accessions taken from across the globe [21].

The sequence identity distribution per TE family relates to the divergence among copies and exhibits their transposition history. These distributions indicate waves of TE invasions suggesting multiple TE invasions by “bursts” of transposition (i.e.*,* numerous transposition events over a short period of time); there were often more than one burst for each TE family over several million years [15].

### Transposable element mobility in Arabidopsis

The LTR retrotransposon ATCOPIA93 (also called Evadé), and the two DNA transposons ATENSPM3 and VANDAL21, are the most mobile in the species [21]. TE copy numbers correlate with climate and/or genetic factors [21]. Nine class I (ATLINE1_2, ATLINE1_5, ATCOPIA2, ATCOPIA31, ATGP2, ATCOPIA78, ATCOPIA28, ATCOPIA45, ATCOPIA89) and six class II TE families (VANDAL20, ATDNA1T9A, ATENSPM2, ATENSPM1, ATMU10, VANDAL11) show correlations with geo-climatic variables. Among them, ATCOPIA78 (also known as ONSEN), displays the strongest correlation with the annual temperature range. The transcription factor ARF23 which recognizes motifs present on the sequences of ATGP2 and ATENSPM2 appears associated with the copy number of these families. MET2a, a poorly characterized homolog of MET1 (the main DNA methyltransferase), is associated with the mobility of a large number of TE families. This gene seems to affect the CHG methylated sites involved in TE repression [22, 23].

Repression of transposition involves a variety of mechanisms, including covalent modifications of histones, DNA methylation, incorporation of histone variants, and other factors, such as chromatin-remodelling enzymes or small RNAs. TE sequences are typically methylated at CG, CHG and CHH sites (where H = A, T or C) in a process that requires numerous factors, including small interfering RNAs (siRNAs) to guide methylation of homologous DNA sequences, and so called de novo and maintenance DNA methyltransferases. Overall, ~ 75% of TEs are methylated [9]. The fraction of methylated sites within TE sequences differs dramatically between CG, CHG and CHH sites, whereas CG sites are all unmethylated or all methylated, the fraction of methylated CHG sites varies almost monotonously between 0 and 100% and that of CHH sites rarely exceeds 50%. TE families with recent transposition activity appeared more methylated [21]. Chromatin state associated with the histone H3K9me2 and H3K27me1 methylation marks are predominantly present on silent TEs [24]. De novo DNA methylation is mediated through the RNA-directed DNA methylation (RdDM) pathway, which involves small interfering RNAs (siRNAs) and scaffold RNAs with proteins [12]. RdDM pathway produces 24-nucleotide siRNAs through RNA POLYMERASE IV (POL IV) transcription followed by RNA-DEPENDENT RNA POLYMERASE 2 (RDRP2 or RDR2) to generate a double-stranded RNA then cleaved by DICER-LIKE PROTEIN 3 (DCL3) into siRNAs. The siRNAs are loaded onto ARGONAUTE (AGO) proteins and pair with complementary nascent transcripts produced by POL V. AGO4 interacts with the DNA methyltransferase DOMAINS REARRANGED METHYLASE 2 (DRM2), to catalyse de novo DNA methylation in a sequence-independent manner. This may be assisted by RNA-DIRECTED DNA METHYLATION 1 (RDM1), which associates with both AGO4 and DRM2 which may bind single-stranded methylated DNA. Where RdDM is inhibited, CMT2 catalyses methylation at histone H1-containing heterochromatin, with DECREASED DNA METHYLATION 1 (DDM1), a chromatin-remodelling protein also required for maintaining DNA methylation in symmetric cytosine sequence contexts. CG and CHG methylation can be maintained during DNA replication by DNA METHYLTRANSFERASE 1 (MET1) and CHROMOMETHYLASE 3 (CMT3). But CHH methylation must be re-established every cell generation, presumably by one of two de novo pathways, one involving CHROMOMETHYLASE 2 (CMT2), the other RNA-directed DNA methylation (RdDM) [12]. CMT2 preferentially methylates heterochromatic non-CG cytosines, while RdDM involves small RNAs.

TEs are generally thought to insert anywhere in the genome, but some families show strong insertion bias. Athila elements are almost exclusively inserted in the pericentromeric regions, whereas other LTR retrotransposons are inserted in progressively less proximal regions of the chromosome arms, the trend being more pronounced for the Gypsy superfamily [17]. No correlation between age and relative distance from centromeres has been found for complete Athila elements. This strongly suggests that these elements have evolved to preferentially target the pericentromeric heterochromatin, and their genomic distribution, unlike that of Copia-like elements, is not the result of a passive accumulation. Some Ty3/gypsy retrotransposons have chromodomains at their integrase C termini. These chromodomains may preferentially target heterochromatin for insertion, because they are able to recognize histone H3K9 methylation marks, an epigenetic mark characteristic of heterochromatin [25]. The potential presence of such chromodomains in *Arabidopsis* Gypsy-like TE families may explain this strange feature.

The preferred substrate for integration of VANDAL21, ATENSPM3, and ATCOPIA93 is the euchromatin [21]. VANDAL21 targets mainly promotors and 5’UTR of broadly active genes which are enriched in histone marks H3K4me3 and H3K36me3. ATENSPM3 and ATCOPIA93 target repressed genes enriched in their body in the histone mark H3K27me3 and the histone variant H2A.Z. ATCOPIA93 is also found overrepresented in gene bodies solely enriched in H2A.Z. Interestingly, loci controlling adaptive responses to the environment are the most frequently observed transposition targets [21].

### Forces and mechanisms known to modulate TE dynamics in Arabidopsis

Two non-exclusive models of TE population dynamics may explain their insertion pattern: the “ectopic recombination” and the “gene-disruption” models [26]. According to the first model, TEs present as dispersed homologous sequences may induce ectopic recombination leading to genome rearrangements (e.g., duplications, deletions and inversions). This model predicts that TE sequences are eliminated due to the deleterious effects of these genome rearrangements. In consequence, TE sequences in high recombination rate regions will be more quickly eliminated [27]. This model also predicts an accumulation of TE sequences in regions with low meiotic recombination rates such as centromeres [28].

In *A. thaliana,* no significant correlation between the TE density and the recombination rate has been found [16, 29], but a significant inverse correlation between the densities of repeats and genes does exist. This indicates that the presence of TEs within or close to genes is deleterious, in favor of the “gene-disruption” model of TE dynamics. Indeed, according to this model, TE insertions into genes or their regulatory regions are strongly selected against. Consequently, repeats accumulate in gene-poor regions. This model appears to provide a good explanation of TE dynamics in the *A. thaliana* genome in contrast to *Drosophila melanogaster* where the accumulation of TE sequences is negatively correlated with recombination rates [30]. This difference may come from the “selfing” mode of reproduction of *Arabidopsis thaliana*. Indeed, in selfing species, ectopic recombination is believed to be rare, as selfing induces homozygosity. TE insertions are then homozygous and for a given allelic position template choice during the recombination process will be driven towards the allelic position on the sister chromatid or the homologous chromosome, preventing ectopic homologous repair. The effect of recombination on TE distribution in selfing species is thus expected to be weak [28].

## Impact on host genes

### TEs as new genes

Sometimes a TE acquires a functional role for the host, and then remains conserved in the genome. In some celebrated cases, TEs have been co-opted to play key organismal functions in *Arabidopsis*. Transposases thus become domesticated by the host to fulfil important cellular functions.

The FHY3 and FAR1 genes encode two proteins related to Mutator-like transposases [31]. They act together to modulate the photoreceptor phytochrome A which mediates various far-red light induced responses. FHY3 and FAR1 both possess separable DNA-binding and transcriptional activation domains that are highly conserved in Mutator-like transposases. It has been shown that they interact with PHYTOCHROME-INTERACTING FACTOR1 to regulate chlorophyll biosynthesis by modulating HEMB1 during de-etiolation in *Arabidopsis* [32].

A transposase called DAYSLEEPER has been shown to be essential for normal plant growth [33]. It shares several characteristics with the hAT family of transposases (hobo, Activator, Tam3), and binds to the Kubox1 motif present in the upstream region of the Arabidopsis DNA repair gene Ku70. This motif appears conserved in the upstream regions of many other plant genes. Plants lacking DAYSLEEPER or strongly overexpressing it do not develop in a normal manner indicating that it is essential for plant development.

A family of domesticated TEs called MUSTANG have been shown to be functional [11]. When mutated, they give rise to phenotypes with severely reduced plant fitness (small plant size, delayed flowering, abnormal development of floral organs, and markedly reduced fertility). This gene family is present in all flowering plants, but not in any non-flowering plant lineages, such as gymnosperms, suggesting that the molecular domestication of this family may have been an integral part of early angiosperm evolution.

### TEs as gene regulators

Many studies show TEs being co-opted into regulatory sequences of genes. Two mechanisms have been described so far.

First, TEs may repress adjacent genes through epigenetic mechanisms. If they are targeted by siRNA and methylated, their repressive chromatin state may affect adjacent gene sequences. The methylation has been shown to spread to adjacent sequences over ~ 300 bp on both sides [9, 21]. This may affect nearby gene expression. Moreover, the methylated adjacent region often remains methylated after deletion of the TEs [34] providing a possible explanation for the presence of some methylated sequences in the absence of TE insertions.

**Fig. 4:**
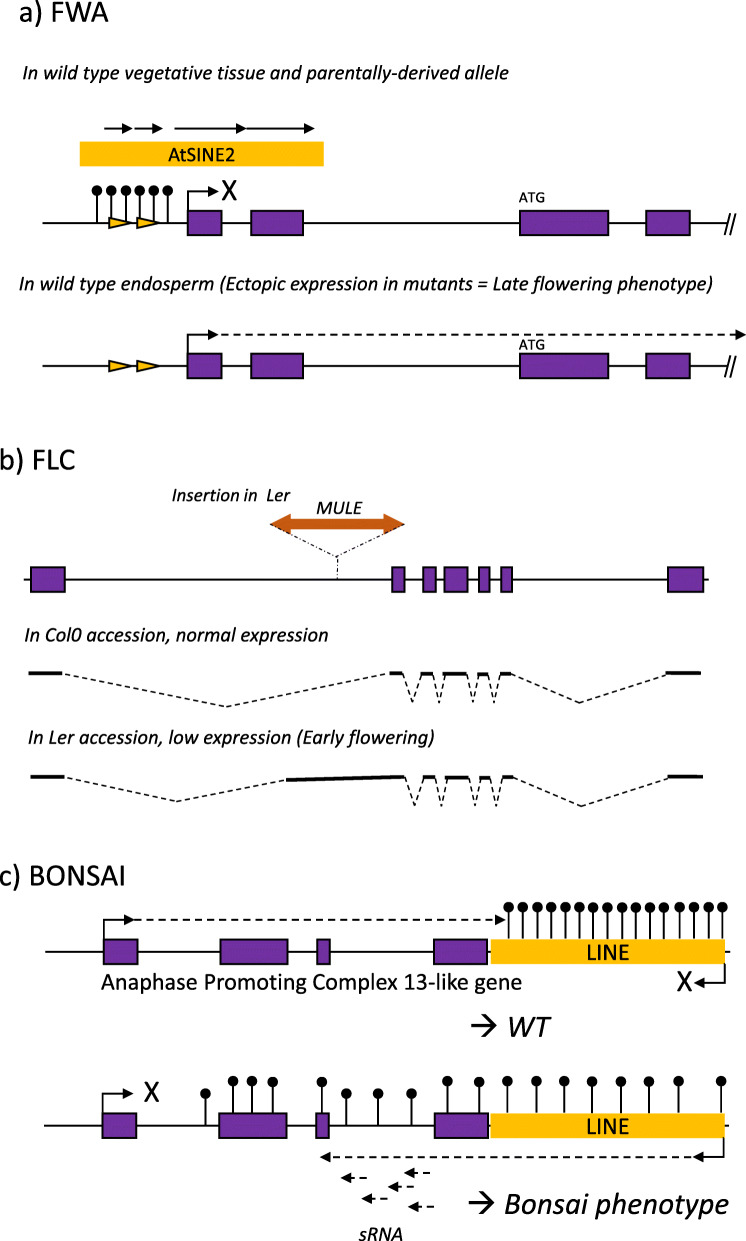
Some examples of TE insertion impact on genes. **a)** The FWA locus is repressed in vegetative tissues and the parentally-derived allele through an epigenetic mechanism induced by an old SINE insertion. Ectopic expression of the locus in a demethylated context causes a late-flowering phenotype. **b)** The FLC locus has a lower expression in the Ler accession caused by a Mutator-like insertion that affects the mRNA structure. **c)** The BONSAI locus is repressed by the loss of methylation of a LINE element inserted downstream. The TE is transcriptionally reactivated and produces small RNAs that repress the APC13-like gene to provoke a Bonsai phenotype

The FWA locus exemplifies this mechanism. This imprinted gene of *Arabidopsis thaliana* is expressed specifically in the endosperm [10, 35]. Its expression depends on the methylation status of its promoter which is similar to a SINE retroelement (Fig. 4a). Methylation of this element causes epigenetic silencing which prevents its expression in vegetative tissues and paternally-derived alleles. In reduced methylation background, the FWA gene has an ectopic expression which leads to a late-flowering phenotype [36].

Another interesting example is the FLOWERING LOCUS C (FLC). This gene is a central repressor of flowering that contributes to natural differences in flowering behaviour among *A. thaliana* accessions. The FLC allele in the Ler accession contains a Mutator-like insertion into an intron (Fig. 4b) resulting in a low expression level, through an unknown mechanism [37, 38]. This TE renders FLC-Ler subject to repressive chromatin modifications mediated by short interfering RNAs generated from homologous transposable elements in the genome [39]. FLC is a candidate for the major-effect QTL underlying variation in the vernalization response: a weak FLC allele caused by a Mutator-like transposon contributes to flowering time variation in two North American accessions [40]. Interestingly, this locus has also been found as target for insertion of new TE copies in natural populations [21]. The authors suggest that they are retained by natural selection for the adaptation to warm climate they provide by reducing transcription of FLC. This low FLC expression would result in earlier flowering providing drought resistance.

In contrast to the FWA and FLC examples, the *bns* (BONSAI) phenotype (characterized by short, compact inflorescence, and a reduced plant height) results from a loss of TE methylation and a gain in TE expression. The transcription of a LINE element in the downstream convergently transcribed gene leads to epigenetic silencing of the Anaphase Promoting Complex (APC) 13-like gene (Fig. 4c) when the LINE is activated [41].

Second, TE may affect gene expression via TE transcription factor binding sites (TFBSs). Indeed, as TEs need to be transcribed to transpose, their sequence also contains TFBSs. When inserted close to a gene, these TFBSs may affect transcription of adjacent genes by recruiting additional transcription factors (TFs). Interestingly, many TEs have been shown to be induced by heat stress. New insertions of these stress sensitive TEs are thought to generate novel stress-responsive regulatory gene networks. Hence, natural and experimentally induced variants of ATCOPIA78/ONSEN insertions confer heat responsiveness to nearby genes [42, 43]. The LTR of ATCOPIA78/ONSEN contains heat-responsive elements [44]. ONSEN, COPIA37, TERESTRA, and ROMANIAT5 are the major families of heat-responsive TEs in *A. lyrata* and *A. thaliana*. The heat-responsive elements of ONSEN are conserved over millions of years and were already present early in the evolution of the *Brassicaceae*.

Another co-option example shows that RC/Helitron TEs have served as distributors of PHE1 DNA-binding sites [45]. PHE1 is a key transcriptional regulator of imprinted genes, type I MADS-box TFs, and genes required for endosperm proliferation and cellularization. This example shows the key role of TEs in establishing a reproductive barrier between individuals of different ploidies through PHE1.

Mechanisms of TE exaptation can be very diverse. A completely different example shows a recent lineage-specific TE exaptation which resulted in the expansion of a core regulon of *Arabidopsis* Trp-derived defense metabolism [46]. A LINE retrotransposon that they called *EPCOT3* has retroduplicated from a WRKY33-TFBS-carrying progenitor and inserted upstream of the newly duplicated gene *CYP82C2*. Chromatin remodeling has led this LINE element to become *a* bona fide enhancer.

A link between the responsiveness of TEs to biotic stresses has also been established in Arabidopsis with the co-option of a soloLTR derived regulatory sequence. Indeed, the LTR of *ATCOPIA93* has been shown to be activated during pathogen defense in *Arabidopsis* [47]. The endogenous *ATCOPIA93* copy “*EVD*” is activated in the presence of bacterial stress as well as a LTR-*GUS* construct. Interestingly, an *ATCOPIA93*-derived soloLTR is found upstream of RPP4, a the disease resistance gene.

Overall a probable large fraction of genes are affected in their expression by the presence of TEs in their proximity [21, 34]. Both repression and activation patterns are observed for TEs inserted upstream of genes, whereas mostly repression is observed for TEs inserted in gene bodies or downstream. The alteration is more pronounced for COPIA elements and less pronounced for MuDR.

Recent population genetic approaches reveal TEs being targets of positive selection. In their study Li et al. [48], identified 33, 7, and 13 adaptive TE candidates in 3 populations. Screening 20 kb regions surrounding these TEs they found 2 adaptive TE candidates, a LINE and a Copia, with higher haplotype homozygosities in TE insertion alleles than alleles without the TEs.

### TEs and exon shuffling

TIR transposons can be potentially mobile as non-autonomous elements. Consequently, if they capture chromosomal fragments, they can disperse it throughout the genome. Models explaining how these TEs could acquire chromosomal DNA remain very speculative. Several authors postulated that their activity can accelerate the genome evolution through exon shuffling. For example, Pack-MULEs populate the rice genome and had a major impact on the current organization of rice chromosomes and the evolution of rice genes [49]. Structures resembling Pack-TYPE transposons are also present in *Arabidopsis* [50, 51] deriving from Mutator-like (MULE) and En/Spm (also known as CACTA), transposon families. Mobile elements with CACTA-derived TIRs show real time mobilization suggesting a new model of gene shuffling [52].

All the results presented above suggest that repeats have profound effects on plant genome biology shaping gene architectures and regulating phenotypic variations.

## Transposable elements in long-term genome evolution

Few studies explore the evolution of repeated sequences over long periods of time. In the *Arabidopsis thaliana* genome, it has been shown that the majority of the repeats found are ancient and likely to derive from the retention of fragments deposited during ancestral transposition events [53]. This analysis found one third more TEs than the current official TE annotation, identifying old TE remnants that probably appeared in bursts early in Brassicaceae evolution, more than 40 Myr ago. Interestingly, TEs specific to *A. thaliana* lineage contribute only 36.8% of the identified TEs, whereas TEs present in common ancestors with *A. lyrata* and *B. rapa* contribute as much as 17.5 and 25.8%, respectively. The majority of recent TEs are found in pericentromeric domains, while older ones are frequent in the gene-rich regions [53, 54].

DNA methylation of repeats through small RNA-mediated pathways can last over prolonged periods of time [53]. Therefore, the mutation process of TE sequences is mainly driven by the deamination of methylcytosines which replaces cytosines by thymines. TE-rich regions tend then to be A/T rich which impacts genome composition and epigenomic landscapes. Hence, TEs and their decayed sequences contribute to the genome bulk. The vast majority of repeated elements accumulate mutations to the extent of becoming anonymous sequences, also known as ‘genomic dark matter’ thought to contribute significantly to the composition of plant genomes.

## Future directions

### TE origin of genes and regulatory sequences

TE annotation is limited by DNA sequence alignment algorithms which require at least ~ 65% nucleotide identity with the TE reference sequence to be considered. Using Jukes and Cantor’s evolutionary model for non-coding sequences, and the molecular clock used for TEs in rice, generally taken as reference for all plants, we obtain a limit at ~ 36 Myr Myr for TE detection (in the absence of selective pressure). At this age, TEs are expected to conserve only 27% of their length if we use a model for continuous decay [55]. Consequently, published results are restricted to timescales that permit TE identification with standard approaches, that would have missed older TEs that are too degraded to be recognized. Hence, current methodology hampers exploration of the impact of TEs at a timescale up to the apparition of flowering plants between − 200 and − 100 Myr during the Jurassic/Cretaceous period. However, looking at this timeline could reveal the role played by TEs during the colonization of earth by flowering plants. Important Gene Regulatory Networks may have appeared at this crucial time, putting in place very fundamental adaptive responses, with the help of some TEs, for most flowering plants. As we have shown in this review, TEs are able to modify gene expression according to environmental conditions. Therefore, they might have played an important role in adaptation, in particular for the success of flowering plants.

Very recently a new approach was designed specifically for finding old and degenerated TE copies [56]. It uses a different strategy with *k-mers* implemented into an alignment-free algorithm. This study reported that half of the *Arabidopsis* genome seems to originate from TEs. Very probably more genes are made of TE parts than was first envisaged. This study also shows a number of TFBSs derived from degenerated TE sequences, suggesting a role of TEs in the regulation of many genes. *Arabidopsis*, given the wealth of available data, thus emerges as a favorable model to study the TE origin and evolution of genes, TFBSs, and promotors in plants.

### TE insertions in the common ancestor of Brassicaceae

Most TE insertions are old and appeared to occur in the common ancestor of Brassicaceae species. A more precise determination of the insertion age of these sequences would allow us to follow the evolutionary history of TE families in the diverse Brassicaceae species. Few studies show the diversification of TE families in a genus, *Arabidopsis* with the number of sequenced related species would allow in-depth investigation of this question.

## Conclusion

The first re-sequenced accessions dramatically improved our knowledge of TE dynamics in this species [21, 34]. But we are still at the beginning of these studies. Today, long-read sequencing can provide access to complete assembled genomes at low cost. This will allow us to study the role and dynamics of TE in pan-genome compartments, i.e. core and dispensable genomes, which will be fundamental to understanding the role of TE in local adaptation. We still have a lot to learn from private, abundant, or fixed insertions, as well as their role in the structure of the dispensable genomes.

Predicting the impact of TE insertion on neighbouring genes would be of tremendous benefit to help understand phenotypic variation among the accessions of this species. TE annotation must go one step beyond what is currently provided. As genes have structural and functional annotations, TE should also have functional annotations in addition to the structure that is currently provided. In particular, TE annotation should indicate TFBS and promotors that are present on each inserted copy to predict the potential functional role exerted on neighbouring genes.

## Data Availability

Not applicable.
